# Nearly Complete Genome Assembly of the Pinewood Nematode Bursaphelenchus xylophilus Strain Ka4C1

**DOI:** 10.1128/MRA.01002-20

**Published:** 2020-10-15

**Authors:** Mehmet Dayi, Simo Sun, Yasunobu Maeda, Ryusei Tanaka, Akemi Yoshida, Isheng Jason Tsai, Taisei Kikuchi

**Affiliations:** a Department of Infectious Diseases, Faculty of Medicine, University of Miyazaki, Miyazaki, Japan; b Forestry Vocational School, Duzce University, Duzce, Turkey; c Laboratory of Genomics, Frontier Science Research Center, University of Miyazaki, Miyazaki, Japan; d Biodiversity Research Center, Academia Sinica, Taipei, Taiwan; Vanderbilt University

## Abstract

Bursaphelenchus xylophilus has been destroying pine forests in East Asia and western Europe. Here, we report its nearly complete genomic sequence containing five ∼12-Mb scaffolds and one ∼15-Mb scaffold representing six chromosomes. Large repeat regions that were previously unidentified are now reasonably integrated, particularly in the ∼15-Mb scaffold.

## ANNOUNCEMENT

Bursaphelenchus xylophilus causes pine wilt disease and has caused extensive damage to pine forests in East Asia and western Europe (1). A 75-Mbp *B. xylophilus* draft genome (v1.2) was previously sequenced using 454 and Illumina GAII technologies with 5,527 scaffolds and an *N*_50_ value of 950 kb (2). Here, we provide a nearly complete genome sequence produced through Nanopore long-read sequencing and Hi-C scaffolding.

The *B. xylophilus* strain Ka4C1, maintained at the University of Miyazaki, was cultured for 7 days on Botrytis cinerea that was grown on autoclaved barley grains. Mixed-stage worms were collected from the culture using the modified Baermann funnel technique (3). Briefly, worm culture was suspended in distilled water (dH_2_O) complemented with streptomycin, amphotericin B, and penicillin (antibiotic/antimycotic [anti/anti]; Gibco), and live worms were passed through a Kimwipe-lined (Crecia) sieve followed by discontinuous sucrose gradient centrifugation to remove culture debris (4). The worms were transferred to a worm lysis solution (Qiagen buffer G2 with 800 µg/ml proteinase K, 50 mM dithiothreitol [Wako], and 0.5 mg/ml RNase A [Invitrogen]) and incubated at 55°C for 4 h after two freeze-thaw treatments. High-molecular-weight genomic DNA was spooled from ethanol precipitation following phenol-chloroform extraction and dissolved in 10 mM Tris (pH 8.0). A Nanopore library was prepared using 1 µg genomic DNA using a ligation sequencing kit SQK-LSK109 (Oxford Nanopore Technologies) according to the manufacturer’s protocol. A single 24-h sequencing run was performed with MinION R9.4.1 flow cells to obtain 2.7 Gbp of sequence data (182,569 reads; *N*_50_, 27 kbp). The Nanopore reads were base called to generate FASTQ files using the Guppy v4.0.15 basecaller (Oxford Nanopore Technologies) with the supplied dna_r9.4.1_450bps_hac configuration and were quality checked using NanoPlot v1.31.0 (5). An Illumina paired-end sequencing library was prepared from 1 µg of DNA using a TruSeq DNA sample preparation kit according to the manufacturer’s instructions. A total of 6.0 Gbp of paired-end reads (100 bp × 2) were generated by library sequencing on an Illumina HiSeq 2000 instrument according to the manufacturer’s protocol. The raw Illumina sequence data were used for generating the genome assembly after removing the adapters and low-quality and duplicate reads using the Real-Time Analysis (RTA) v1.12.4.2 analysis pipeline (Illumina). The Hi-C library was prepared from ∼10,000 fresh worms using an Arima-HiC kit (Arima Genomics) and a Collibri ES DNA library prep kit (Thermo Fisher Scientific) according to the manufacturers’ protocols and was sequenced using a MiSeq instrument with the MiSeq reagent kit v3 (101 cycles × 2), and the 3.8 million short reads were quality checked using the Hi-C quality control pipeline (https://phasegenomics.github.io/2019/09/19/hic-alignment-and-qc.html).

The Nanopore long reads were assembled using Flye v2.7.1 (6) with the parameters –genome-size 75M and –iteration 4. After base correction by two rounds of Pilon v1.23 (7) with the Illumina paired-end reads, the assembly was further scaffolded using the 3D-DNA pipeline v180114 (8) without a misjoin correction process, and the chromosome-length scaffolds were extracted via manual curation using Juicebox v1.11.08 (9).

The final assembly was 78.3 Mbp long, which is 3.7 and 2.4 Mbp bigger than the previous v1.2 (2) and v2.0 (10) assemblies, respectively, and showed slightly better CEGMA v2.5 (11) completeness values (Table 1). However, a G+C content of 40.4% was consistent for all assemblies. The new assembly contains six large scaffolds (five ∼12-Mbp and one ∼15-Mbp scaffolds) representing six chromosomes and five unassigned <20-kb contigs, giving an *N*_50_ value of 12.8 Mbp. The largest scaffold contains large repeat sequence regions, which were unidentified in previous assemblies. The assembly stats of the old and new versions are summarized in Table 1.

**TABLE 1:** Genome stats of published (v1.2 and v2.0) and new (v5.0) assemblies of *Bursaphelenchus xylophilus*

Statistic	Data for genome version:		
	v1.2	v2.0	v5.0
BioProject accession no.	PRJEA64437	PRJDB7519	PRJEB40022
Main sequencing technologies^*a*^	454, Illumina PE	Illumina PE, MP	Nanopore, Hi-C
Assembly size (Mbp)	74.6	75.9	78.3
No. of scaffolds/chromosomes	5,527	501	11
*N*_50_ (kb)	949	1,577	12,794
*L*_50_ (no.)	22	18	3
*N*_90_ (kb)	4.3	344.2	12,140
*L*_90_ (no.)	98	57	6
Maximum length of scaffold/chromosome (kb)	3,612	4,118	15,101
G+C content (%)	40.4	40.4	40.4
CEGMA completeness (%) (complete/partial)	97.8/98.0	97.6/98.4	98.0/98.8
Avg CEG gene no. (complete/partial)	1.08/1.09	1.18/1.23	1.12/1.16

a PE, paired end; MP, mate pair.

### Data availability.

The *B. xylophilus* v5 assembly was deposited in DDBJ/EMBL/ENA/GenBank under project PRJEB40022. The raw Illumina, Nanopore, and Hi-C read data are available in the DDBJ Sequence Read Archive under the accession numbers DRR067231, DRR243685, and DRR243686, respectively.
